# Supplementary material for: Predicting chemical shifts with graph neural networks
Source: Chem Sci. 2021 Jul 9;12(32):10802–9. doi: 10.1039/d1sc01895g (PMC8372537; doi:10.1039/d1sc01895g)
Supplement: SC-012-D1SC01895G-s001 [file SC-012-D1SC01895G-s001.pdf]

Electronic Supplementary Material (ESI) for Chemical Science.
This journal is © The Royal Society of Chemistry 2021

Predicting Chemical Shifts with Graph Neural Networks

Ziyue Yang, Maghesree Chakraborty and Andrew D White

1 Model Stats

Table 1. Complete model stats on test data across classes and atom types for Model (all) Metabolome.

title	corr-coeff	R^2	MAE	RMSD	N
overall	0.8723	0.7609	0.778	1.12	699
overall-H	0.8723	0.7609	0.778	1.12	699
class/MB	0.8723	0.7609	0.778	1.12	699
names/H	0.8723	0.7609	0.778	1.12	699

Table 2. Complete model stats on test data across classes and atom types for Model (all).

title	corr-coeff	R^2	MAE	RMSD	N
overall	0.9997	0.9994	0.7988	1.4570	65163
overall-N	0.9183	0.8432	2.1260	2.9820	7265
overall-C	0.9997	0.9993	1.1190	1.6520	25378
overall-H	0.9916	0.9832	0.2522	0.3676	32520
class/ALA	0.9998	0.9996	0.8593	1.3090	3307
class/ARG	0.9995	0.9991	0.7814	1.6900	3454
class/ASN	0.9997	0.9993	0.9936	1.5960	2319
class/ASP	0.9997	0.9994	0.8585	1.4270	3158
class/CYS	0.9996	0.9992	1.1620	1.7840	680
class/GLU	0.9997	0.9995	0.7212	1.3250	4368
class/GLN	0.9997	0.9994	0.8434	1.4370	2458
class/GLY	0.9997	0.9994	0.8926	1.4930	2956
class/HIS	0.9987	0.9974	1.2830	3.0880	1112
class/ILE	0.9997	0.9994	0.6935	1.2470	5415
class/LEU	0.9998	0.9995	0.6386	1.1040	7948
class/LYS	0.9998	0.9995	0.6461	1.1720	6018
class/MET	0.9996	0.9993	0.9166	1.5220	1124
class/PHE	0.9994	0.9989	1.1840	2.2350	3546
class/PRO	0.9999	0.9997	0.5865	0.9146	2463
class/SER	0.9997	0.9993	0.8792	1.5200	2683
class/THR	0.9997	0.9994	0.8144	1.3980	3412
class/TRP	0.9995	0.9990	1.1910	1.9550	982
class/TYR	0.9997	0.9994	0.9679	1.5110	2068
class/VAL	0.9998	0.9995	0.6532	1.1990	5692
names/N	0.8795	0.7735	2.1070	2.7540	6822
names/C	0.8268	0.6836	0.9708	1.2880	6012
names/H	0.7180	0.5156	0.3914	0.5274	6859
names/CA	0.9704	0.9418	0.9562	1.2600	7385

Continued on next page

Table 2. Complete model stats on test data across classes and atom types for Model (all).

title	corr-coeff	R^2	MAE	RMSD	N
names/CB	0.9916	0.9832	1.1640	1.7620	6069
names/CG	0.9990	0.9980	0.9336	1.3190	1513
names/CD	0.9997	0.9993	0.8187	1.1310	666
names/HA	0.8442	0.7126	0.2185	0.2931	5031
names/HB2	0.9049	0.8189	0.2320	0.3212	3105
names/HB3	0.9012	0.8122	0.2405	0.3372	2950
names/HG2	0.8538	0.7289	0.1823	0.2648	1202
names/HG3	0.8619	0.7429	0.1934	0.2683	1094
names/HD2	0.9887	0.9776	0.2185	0.3308	957
names/HD3	0.9491	0.9008	0.2024	0.3000	578
names/CG1	0.9065	0.8218	0.9525	1.4850	548
names/CG2	0.8283	0.6861	1.1040	1.4470	783
names/CD1	0.9996	0.9991	1.4960	1.9270	886
names/HB	0.9521	0.9066	0.2180	0.3381	881
names/HG12	0.5911	0.3494	0.1584	0.2102	323
names/HG13	0.6414	0.4114	0.1496	0.1990	323
names/HG21	0.6602	0.4358	0.1821	0.2561	870
names/HG22	0.6827	0.4661	0.1834	0.2457	870
names/HG23	0.6720	0.4516	0.1804	0.2471	870
names/HD11	0.6201	0.3846	0.1715	0.2323	695
names/HD12	0.6111	0.3735	0.1754	0.2340	695
names/HD13	0.6438	0.4145	0.1712	0.2283	695
names/HG11	0.6327	0.4003	0.1536	0.2012	323
names/NE	0.3560	0.1267	2.2390	6.7040	80
names/CZ	0.9592	0.9200	3.6790	5.2430	117
names/HE	0.4747	0.2254	0.3819	0.6412	85
names/CE	0.9835	0.9673	1.1730	1.8420	309
names/HE2	0.9922	0.9845	0.1785	0.2535	519
names/HE3	0.9877	0.9756	0.1542	0.2466	265
names/HZ2	0.2921	0.0853	0.3249	0.4492	43
names/HZ3	0.2396	0.0574	0.2974	0.3879	34
names/CD2	0.9994	0.9988	1.8970	2.4050	559
names/HG	0.7605	0.5784	0.2571	0.3933	348
names/HD21	0.6099	0.3720	0.1767	0.2317	387
names/HD22	0.6594	0.4349	0.1699	0.2232	387
names/HD23	0.6513	0.4242	0.1646	0.2178	387
names/CE1	0.8876	0.7879	3.6570	4.8930	240
names/CE2	0.8720	0.7604	3.8760	4.8920	161
names/HD1	0.4631	0.2144	0.2597	0.3491	351
names/HE1	0.9070	0.8227	0.3349	0.4816	375
names/HA2	0.4050	0.1640	0.3598	0.4711	428
names/HA3	0.2882	0.0830	0.3131	0.4471	417
names/HZ	0.4820	0.2324	0.2967	0.3927	123
names/NE2	0.9710	0.9429	2.1670	3.9190	136
names/ND2	0.3097	0.0959	2.1160	2.7010	164
names/NE1	-0.0418	0.0017	1.8260	2.2530	53
names/CE3	-0.0315	0.0010	2.8640	3.4630	29
names/CZ2	-0.0507	0.0026	2.5580	2.9830	37
names/CZ3	0.2935	0.0861	3.3860	4.1680	29
names/CH2	0.0438	0.0019	4.2180	4.7470	35
names/HH2	0.0501	0.0025	0.2715	0.3462	40

Table 3. Complete model stats on test data across classes and atom types for Model (H).

title	corr-coeff	R^2	MAE	RMSD	N
overall	0.9935	0.9870	0.2218	0.3235	32520
overall-H	0.9935	0.9870	0.2218	0.3235	32520
class/ALA	0.9867	0.9736	0.2414	0.3311	1046
class/ARG	0.9925	0.9850	0.2149	0.3210	1747
class/ASN	0.9894	0.9789	0.2575	0.3556	946
class/ASP	0.9891	0.9783	0.2489	0.3615	1430
class/CYS	0.9812	0.9627	0.3290	0.4789	291
class/GLU	0.9943	0.9887	0.1926	0.2793	2144
class/GLN	0.9925	0.9850	0.2178	0.3159	1108
class/GLY	0.9812	0.9627	0.3141	0.4281	1391
class/HIS	0.9859	0.9720	0.2923	0.3871	521
class/ILE	0.9952	0.9905	0.1974	0.2768	2917
class/LEU	0.9945	0.9890	0.2055	0.2803	4557
class/LYS	0.9938	0.9877	0.1721	0.2653	3241
class/MET	0.9905	0.9811	0.2489	0.3616	524
class/PHE	0.9846	0.9694	0.2612	0.3600	1812
class/PRO	0.9491	0.9009	0.2328	0.3404	1273
class/SER	0.9822	0.9647	0.2513	0.3802	1193
class/THR	0.9908	0.9817	0.2198	0.3895	1683
class/TRP	0.9831	0.9664	0.2897	0.4129	485
class/TYR	0.9875	0.9752	0.2448	0.3327	1047
class/VAL	0.9954	0.9907	0.1928	0.2744	3164
names/H	0.7803	0.6089	0.3389	0.4596	6859
names/HA	0.8790	0.7727	0.1935	0.2628	5031
names/HB2	0.9219	0.8499	0.2120	0.2917	3105
names/HB3	0.9207	0.8477	0.2196	0.3051	2950
names/HG2	0.8728	0.7618	0.1580	0.2468	1202
names/HG3	0.8902	0.7925	0.1676	0.2395	1094
names/HD2	0.9913	0.9826	0.1912	0.2911	957
names/HD3	0.9651	0.9315	0.1680	0.2476	578
names/HB	0.9609	0.9233	0.1993	0.3088	881
names/HG12	0.6101	0.3723	0.1459	0.1954	323
names/HG13	0.6807	0.4634	0.1348	0.1798	323
names/HG21	0.7792	0.6072	0.1555	0.2090	870
names/HG22	0.7626	0.5815	0.1593	0.2145	870
names/HG23	0.7582	0.5748	0.1573	0.2162	870
names/HD11	0.7086	0.5021	0.1576	0.2054	695
names/HD12	0.6769	0.4582	0.1633	0.2139	695
names/HD13	0.6985	0.4880	0.1569	0.2075	695
names/HG11	0.6818	0.4649	0.1353	0.1799	323
names/HE	0.5674	0.3219	0.3463	0.6170	85
names/HE2	0.9939	0.9879	0.1541	0.2190	519
names/HE3	0.9904	0.9809	0.1409	0.2225	265
names/HZ2	0.7137	0.5093	0.2223	0.2962	43
names/HZ3	0.3677	0.1352	0.2335	0.3057	34
names/HG	0.8169	0.6673	0.2256	0.3578	348
names/HD21	0.7159	0.5126	0.1503	0.1992	387
names/HD22	0.7206	0.5193	0.1488	0.1962	387
names/HD23	0.6993	0.4891	0.1566	0.2038	387
names/HD1	0.6081	0.3698	0.2196	0.2995	351
names/HE1	0.9349	0.8740	0.2689	0.4067	375
names/HA2	0.5470	0.2992	0.2699	0.3631	428
names/HA3	0.4195	0.1760	0.3237	0.4448	417
names/HZ	0.6896	0.4755	0.2259	0.3129	123
names/HH2	0.4505	0.2029	0.1938	0.2504	40
